# Impaired ΔNp63 expression associates with reduced *β*-catenin and aggressive phenotypes of urothelial neoplasms

**DOI:** 10.1038/sj.bjc.6600764

**Published:** 2003-03-04

**Authors:** F Koga, S Kawakami, J Kumagai, T Takizawa, N Ando, G Arai, Y Kageyama, K Kihara

**Affiliations:** 1 Department of Urology and Reproductive Medicine, Graduate School, Tokyo Medical and Dental University, 1-5-45 Yushima, Bunkyo-ku, Tokyo 113-8519, Japan; 2 Department of Pathology, Tokyo Medical and Dental University, 1-5-45 Yushima, Bunkyo-ku, Tokyo 113-8519, Japan

**Keywords:** carcinogenesis, urothelium, p63, *β*-catenin

## Abstract

*p63*, a homologue of the *p53* gene, is considered to be essential for the normal development of stratified epithelia including urothelium. To examine possible roles of p63 in urothelial tumorigenesis, p63 expression was systematically examined in normal urothelium, low-grade papillary noninvasive (LPN) urothelial tumours, and high-grade or invasive carcinomas, using either an isoform-nonspecific or a ΔN-isoform-specific antibody. Expression profiles of p63 were also analysed in cultured cells. Immunoreactivity with the two antibodies was virtually identical in tissue samples examined. Basal and intermediate cell layers of normal urothelium showed intense nuclear p63 immunostaining. This normal staining pattern was preserved in a majority of LPN tumours, whereas it was frequently impaired in high-grade or muscle-invasive carcinomas. At the mRNA level, ΔNp63 expression predominated over TAp63, and amounts of ΔNp63 mRNA correlated with p63 immunoreactivity, confirming that ΔNp63 accounts for p63 expressed in urothelial tissues. In cultured cells, ΔNp63 was also expressed in low-grade tumour cells as well as normal urothelial cells, but undetectable in high-grade aggressive carcinoma cells. Interestingly, impaired ΔNp63 expression significantly associated with reduced *β*-catenin expression that was possibly related to progression of urothelial neoplasms. Thus, impaired ΔNp63 expression characterises aggressive phenotypes of urothelial neoplasms.

In clinical management, it is important to recognise two major variants of urothelial neoplasms (Lee and Droller, 2000). The first variant, which constitutes approximately 70% of the neoplasms, presents as low-grade papillary lesions that usually are confined to the mucosa. This variant is often multifocal and recurs, but has limited potential to progress to muscle-invasive stage (Grossman, 1996; Lee and Droller, 2000). The 5-year survival rate of this variant can reach 90% if treated appropriately. The second variant, which accounts for 30% of urothelial neoplasms, presents as high-grade invasive disease at diagnosis and has a high risk to develop incurable distant metastases (Steinberg *et al*, 1992). Extensive clinicopathologic studies suggest that the majority of invasive carcinomas do not originate in papillary noninvasive lesion (Brawn, 1982), but in flat carcinoma *in situ* (CIS) or can arise *de novo* (Schalken *et al*, 1992; Hudson and Herr, 1995). Accumulating molecular analyses indicate that urothelial neoplasm develops via two distinctive carcinogenesis pathways (Cairns *et al*, 1991; Spruck *et al*, 1994; Orlow *et al*, 1995; Lee and Droller, 2000). Low-grade papillary noninvasive (LPN) tumours are frequently associated with deletions of chromosome 9 and defects of p16, a cyclin-dependent kinase inhibitor, but not with mutations of *p53* tumour suppressor gene (Spruck *et al*, 1994; Orlow *et al*, 1995). In contrast, invasive urothelial carcinomas and CIS are primarily associated with dysfunction of p53 and pRb (Cairns *et al*, 1991; Spruck *et al*, 1994). Thus, urothelial neoplasms appear to develop and progress via at least two distinct pathways with different biological behaviour and clinical outcome; the papillary noninvasive and CIS/invasive pathways.

*p63* is a homologue of the *p53* tumour suppressor gene located at 3q27–3q29 and is also known as *p51*, *p40*, *p73L*, or *KET* (Schmale and Bamberger, 1997; Osada *et al*, 1998; Senoo *et al*, 1998; Trink *et al*, 1998; Yang *et al*, 1998). This gene encodes for two major classes of protein: those containing an acidic amino terminus analogous to the transactivating domain of p53 (TAp63), and those with a truncated amino terminus that lacks this region (ΔNp63). In addition, alternative splicing at the carboxyl terminus yields at least three p63 isoforms (*α*, *β*, and *γ*) (Osada *et al*, 1998; Yang *et al*, 1998). TAp63 isoforms can transactivate p53 target genes such as *p21*, and induce apoptosis when overproduced (Osada *et al*, 1998; Yang *et al*, 1998), whereas ΔNp63 isoforms potentially suppress transactivation by both p53 and TAp63 isoforms in a dominant-negative manner (Yang *et al*, 1998).

Although the function of the *p63* is not fully understood, the striking epithelial defects throughout the body seen in *p63* knockout mice (Mills *et al*, 1999; Yang *et al*, 1999) suggest that this gene plays a key role in maintaining basal, progenitor cell populations of the stratified epithelia (Yang *et al*, 1999; Yang and McKeon, 2000). In fact, p63 is selectively expressed in the basal cell or progenitor cell compartment of stratified epithelia including urothelium in human tissues (Yang *et al*, 1998,^1999^; Di Como *et al*, 2002). Moreover, genitourinary abnormalities have been documented in patients with the ectrodactyly, ectodermal dysplasia, and cleft palate syndrome caused by heterozygous germline mutations of *p63* (Rollnick and Hoo, 1988; Celli *et al*, 1999). Based on these findings, p63 may play critical roles in the normal development and maintenance of the human urothelium.

Despite the initial enthusiasm surrounding the cloning of *p63* as a homologue of *p53* tumour suppressor gene, mutational analyses have demonstrated rare *p63* mutations (<4%) in a large number of human primary tumours including urothelial tumours (Osada *et al*, 1998; Hagiwara *et al*, 1999; Sunahara *et al*, 1999; Park *et al*, 2000). Therefore, *p63* is unlikely to be a tumour suppressor gene that conforms to the two-hit model of carcinogenesis. On the other hand, ΔNp63 is considered to have oncogenic properties based on the following observations: (1) ΔNp63 overexpression is often observed (Parsa *et al*, 1999; Hibi *et al*, 2000) and it enhances oncogenic growth in squamous cell carcinomas (Hibi *et al*, 2000); (2) ΔNp63 could function as dominant negatives against the p53 tumour suppressor activities (Yang *et al*, 1998); and (3) ΔNp63 overexpression induces nuclear accumulation of *β*-catenin and activates the *β*-catenin signalling that promotes cell proliferation (Patturajan *et al*, 2002).

The current study was designed to assess the possible roles of altered p63 expression in the development of urothelial neoplasms. We systematically analysed p63 expression profiles in normal urothelium and urothelial neoplasms of various pathologic phenotypes using tissue samples and cultured cells. This study demonstrates that LPN tumours highly preserve the normal p63 expression pattern characterised by abundant and well-organised ΔNp63 expression, while high-grade/invasive urothelial carcinomas frequently show a decrease or loss of ΔNp63 expression. Furthermore, diminished ΔNp63 expression associates with reduced expression of *β*-catenin that plays important roles in cell adhesion and signal transduction (Conacci-Sorrell *et al*, 2002).

## MATERIALS AND METHODS

### Tissue samples

Seventy-two urothelial tumour specimens (obtained from 61 patients) and five normal urothelium specimens (from five patients with renal cell carcinoma) were used for immunohistochemistry. For the analysis of mRNA, three normal and 32 neoplastic urothelial tissues corresponding to those for immunohistochemistry were snap-frozen in ISOGEN (Wako Co., Osaka, Japan) immediately after the surgical removal and stored at −80°C. Histologic grade of the tumour was determined according to the 1973 World Health Organization grading system (Mostofi, 1973) by two pathologists (JK and TT), without information on p63 expression status. Tumours composed of grades 1–2 cells were classified as low-grade, while those containing grade 3 cells as high-grade. The stage of the tumour was determined based on the 1997 TNM system (Sobin and Wittekind, 1997). Informed consent was obtained from each patient before commencing experiments.

### Human cell lines

To analyse p63 expression in normal human urinary tract *in vitro*, normal human urothelial (NHU) cells and normal human ureteral stromal cells were cultivated as reported elsewhere (Kawakami *et al*, 2002). Also examined were four established human bladder cancer cell lines: RT-4, JTC30, EJ, and T24. RT-4 was derived from papilloma of the urinary bladder (Rigby and Franks, 1970). JTC30 is a cell line established in our laboratory from a patient with well-differentiated papillary bladder tumour (Kakuya *et al*, 1983). EJ was established from invasive, poorly differentiated urothelial carcinoma of the bladder (Evans *et al*, 1977; Hastings and Franks, 1983). T24 was derived from a primary lesion of grade 3 carcinoma of the bladder (Bubenik *et al*, 1973).

### Immunohistochemistry

Immunostaining was performed in paraffin-embedded tissue specimens using a mouse anti-human p63 monoclonal antibody, 4A4 (Santa Cruz Biotechnology, Santa Cruz, CA, USA), which recognises all six p63 isoforms (Yang *et al*, 1998,^1999^). Five-micrometre sections were deparaffinised, rehydrated, and treated in 0.3% hydrogen peroxide in methanol for 30 min to exhaust endogenous peroxidase activity. The sections were pretreated in a 550 W microwave oven three times for 5 min in 10 mM citrate buffer (pH 6.0). After blocking of nonspecific binding with 10% goat serum, sections were incubated with the 4A4 antibody at a 1 : 200 dilution for 1 h at room temperature. After washing with phosphate-buffered saline, the sections were incubated with a horseradish peroxidase-labelled secondary antibody (HISTOFINE Simple Stain MAX PO(M), Nichirei, Tokyo, Japan) for 30 min at room temperature. Peroxidase activity was localised using 3,3-diaminobenzidine (Nichirei, Tokyo, Japan). Standardised development time periods allowed accurate comparison of all samples. Finally, the sections were counterstained slightly with haematoxylin, dehydrated, and mounted. In negative controls, the primary antibody was substituted for nonimmune mouse sera. Nonneoplastic prostate glands were used as positive controls (Signoretti *et al*, 2000). The slides were independently reviewed by two of the authors (KF and KS) who were blinded to the clinicopathologic data. Immunoreactivity was evaluated for staining intensity (none, weak, intense) and extent (none, <10%; heterogeneous, 10–50%; homogeneous, ⩾50%) of each tumour. Immunostaining using a rabbit anti-p40 polyclonal antibody Ab-1 (Oncogene Research Products, Boston, MA, USA; at 1 : 2000 dilution) was also performed to specifically detect ΔNp63 (Hibi *et al*, 2000). Unless stated otherwise, the 4A4 antibody was used in all protein study.

p53 and *β*-catenin expression was examined using mouse monoclonal antibodies, PAb1801 (Oncogene Science, Cambridge, MA, USA; at 1 : 50 dilution) and clone 14 (Transduction Laboratories, Lexington, KY, USA; at 1 : 250 dilution), respectively. Nuclear p53 staining in at least ⩾10% of tumour cells was considered positive, as described previously (Koga *et al*, 2000). This criterion was based on the report in which the *p53* gene mutations strongly correlated with positive nuclear staining in ⩾10% of tumour cells when the PAb1801 was used (Esrig *et al*, 1993). For *β*-catenin expression, the staining pattern similar to that in the normal urothelium (homogeneously positive staining at the cell membrane) was evaluated as normal. Negative and heterogeneously positive staining of the cell membrane was described as reduced. Nuclear immunoreactivity was also evaluated separately from membranous immunoreactivity.

### Reverse transcription–polymerase chain reaction (RT–PCR)

Total RNA extraction from tissue homogenates or cell lysates, and first-strand cDNA synthesis was performed as previously reported (Kawakami *et al*, 2001b). Levels of TAp63 and ΔNp63 mRNAs were estimated by semiquantitative RT–PCR-based assay with hypoxanthine phosphoribosyltransferase 1 (HGPRT) served as an internal control as previously described (Kawakami *et al*, 2001b). Oligonucleotide primers (Table 1

**Table 1:** Sequences, amplicon sizes, and exon localisations of PCR primers

**Primer name**	**Sequence**	**Exon**	**GenBank accession no.**
*TAp*63 *amplicon size*: 296 *bp*			AF 075430
F133	GGTGCGACAAACAAGATTGAG (nt 133–153)	ex3	
R426	GAAGGACACGTCGAAACTGTG (nt 406–426)	ex4	
			
Δ*Np*63 *amplicon size*; 252 *bp*			AF 075431
F12	GGAAAACAATGCCCAGACTC (nt 12–31)	ex3′	
R426	GAAGGACACGTCGAAACTGTG (nt 241–261)	ex4	

) were designed based upon the reported human TAp63 and ΔNp63 cDNA sequences (GenBank Accession numbers AF 075430 and AF 075431, respectively) to span an intron to control for amplification of genomic DNA carryover as described elsewhere (Kawakami *et al*, 2001a). No detectable amplification from genomic DNA was observed with these primers. cDNA fragments corresponding to TAp63 and ΔNp63 mRNAs were amplified by PCR using primer pairs consisting of a shared common downstream primer (R426) and distinct upstream primers unique for each first exon but with similar melting temperature. These primers were also designed to give RT–PCR products with similar size, allowing us to compare TAp63 and ΔNp63 transcript levels with a reasonable accuracy. Optimum PCR conditions to amplify the HGPRT, TAp63, and ΔNp63 cDNAs with specific primers were: 94°C for 5 min for initial denaturation, followed by 30 cycles of 94°C for 30 s, 60°C for 30 s, and 72°C for 60 s. Preliminary assays were performed to optimise the number of PCR cycles so that those products were within the exponential phase of amplification. PCR products were visualised and quantified as described previously (Kawakami *et al*, 2001b).

### Immunoblot analysis

Whole cell lysates were prepared in lysis buffer and separated in 12% sodium dodecyl sulphate–polyacrylamide gel electrophoresis followed by transfer to polyvinylidene difluoride membranes (Immobilon; Millipore, Bedford, MA, USA) as described previously (Kawakami *et al*, 2001b). The membranes were blocked overnight with 5% nonfat dried milk and incubated with the 4A4 antibody for 1 h at room temperature. Immunoreactive bands were visualised as reported elsewhere (Kawakami *et al*, 2002).

### Statistical analysis

The Fisher's exact test or *χ*^2^ test was used to assess the significance of proportions. The Spearman's rank correlation test for analysis of nonparametric data was used to test the correlation between variables. Differences were considered significant when the *P*-value was <0.05.

## RESULTS

### Expression of p63 protein in tissue samples

**Figure 1:**
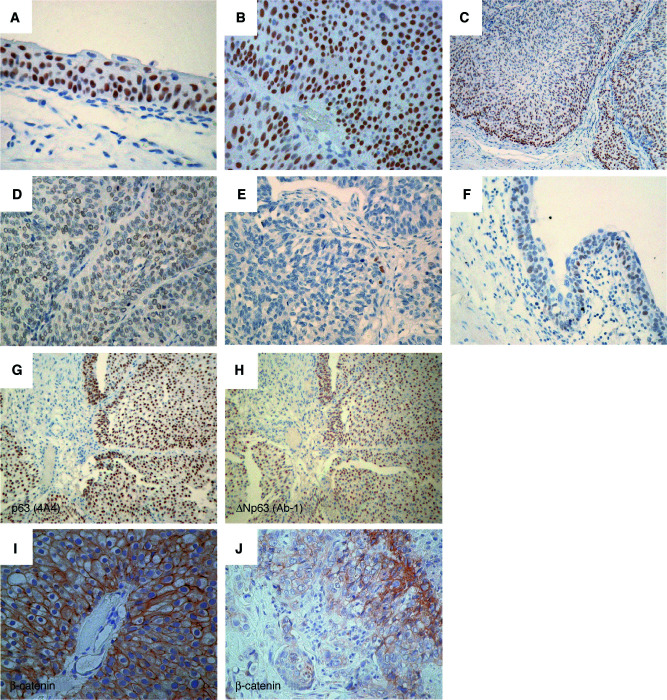
Representative photomicrographs of immunostaining for p63, ΔNp63, and *β*-catenin in urothelial tissues. (**A**–**F**) Immunostaining of p63 with the 4A4 antibody that recognises all isoforms of p63: (**A**) intense staining in normal urothelium (× 200), (**B**) intense staining in an LPN tumour (× 200), (**C**) gradual diminution of p63 expression towards the superficial cell layers in an LPN tumour (× 100), (**D**) weak staining in a high-grade muscle-invasive carcinoma (× 200), (**E**) loss of staining in a high-grade muscle-invasive carcinoma (× 200), and (**F**) weak staining in CIS (× 200). (**G** and **H**) Immunoreactivity with the 4A4 antibody (**G**) is virtually identical to that with the ΔNp63-specific antibody Ab-1 (**H**) (× 100). (**I** and **J**) Immunostaining of *β*-catenin: (**I**) normal *β*-catenin expression with homogeneously positive staining at the cell membrane preserved in an LPN tumour (× 200) and (**J**) reduced (heterogeneously positive) *β*-catenin expression in a muscle-invasive carcinoma (× 200).

We initially evaluated p63 protein expression on normal urothelium and urothelial tumour tissues. Immunoreactivity for p63 was localised to nuclei of normal and neoplastic urothelial cells. Except the urothelium, normal tissues of the kidney, ureter, and urinary bladder were negative for p63. In normal urothelium, almost entire basal and intermediate cells (4–5 layers from the basement membrane) showed intense staining, but immunoreactivity was undetectable in umbrella cells (Figure 1A

).

**Figure 2:**
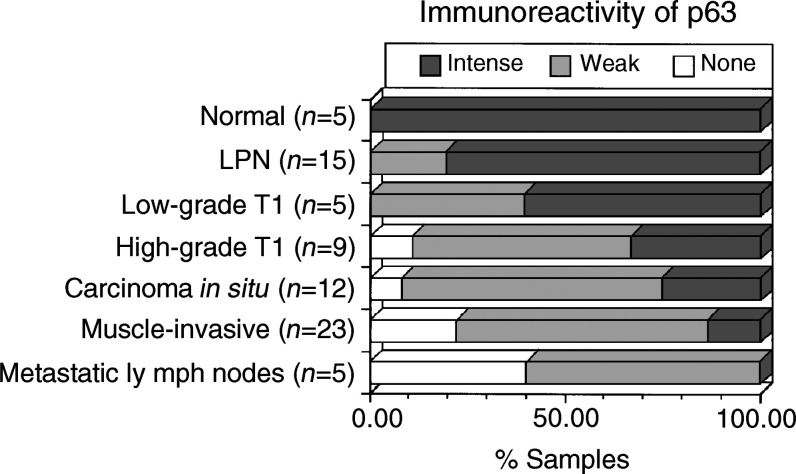
Immunoreactivity of p63 in normal urothelium and urothelial neoplasms of various pathologic phenotypes. All three samples of high-grade Ta tumours showed weak staining.

Immunoreactivity of p63 according to pathologic phenotypes of urothelial tumours is summarised in Figure 2

. All the LPN tumours were positive for p63. Twelve of 15 LPN tumours showed intense p63 staining (Figure 1B and Figure 2). In nine of them, immunoreactivity was strong in basal to middle cell layers as observed in normal urothelia; a gradual diminution of immunoreactivity towards the superficial cell layers was observed (Figure 1C). In contrast, p63 immunoreactivity was weak (Figure 1D) or none (Figure 1E) in most of muscle-invasive (⩾T2) carcinoma tissues. Metastatic lymph nodes demonstrated p63 immunoreactivity identical to that of their primary lesions (weak in three samples and none in two). Six CIS associated with muscle-invasive carcinomas also showed identical immunoreactivity to their primary lesions (intense in one, weak in four and none in one; Figure 1F). Immunoreactivity of p63 in CIS was also similar to that in muscle-invasive carcinomas (Figure 2).

In stroma-invasive (T1) tumours, p63 immunoreactivity was heterogeneous and dependent on histologic grade of the tumour; low-grade tumours showed a slightly lower incidence of intense staining than LPN tumours, whereas a majority of high-grade carcinomas showed weak or undetectable immunoreactivity, similar to muscle-invasive carcinomas (Figure 2). Intense p63 staining was observed in significantly smaller number of primary tumour samples in high-grade (8 out of 41) or muscle-invasive carcinomas (3 out of 23) than in low-grade (15 out of 20, *P*<0.0001) or Ta–T1 tumours (18 out of 32, *P*<0.01), respectively.

We also performed immunostaining using the ΔNp63-specific antibody Ab-1 on samples with positive p63. The staining patterns with the Ab-1 were almost indistinguishable from those with the 4A4 in all samples examined (Figure 1G, H).

### Predominant expression of ΔNp63 mRNA in tissue samples

**Figure 3:**
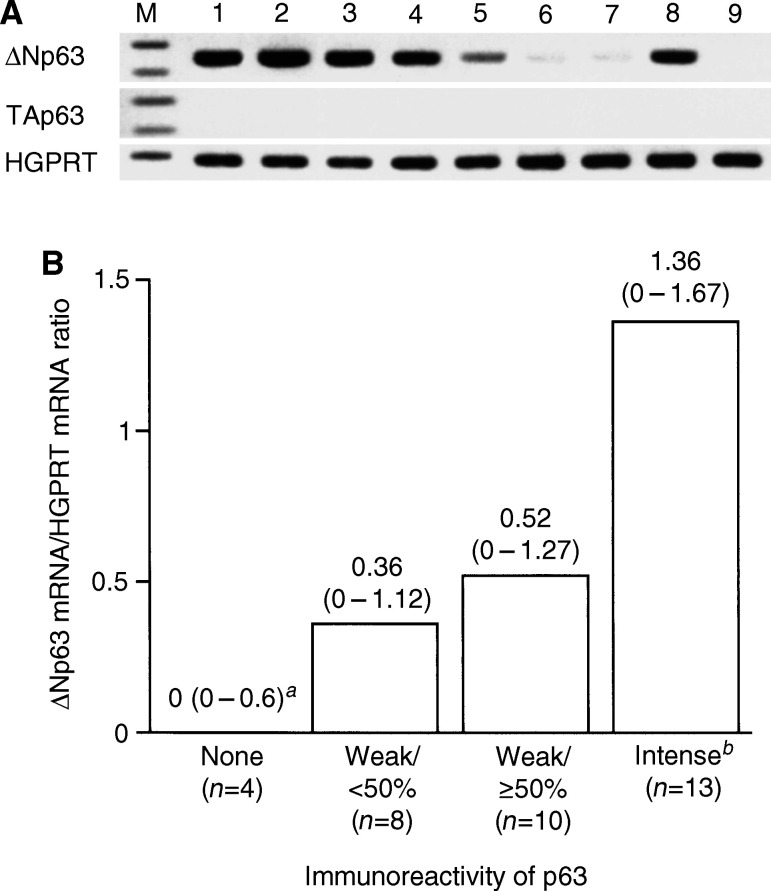
Expression of p63 mRNA in normal and neoplastic urothelial tissues by RT–PCR analysis using isoform-specific primer sets. (**A**) A representative photograph of agarose gel electrophoresis of RT–PCR products. Lane 1, normal urothelium; lanes 2–4, LPN tumours; lane 5, high-grade T1 carcinoma; lanes 6–8, high-grade muscle-invasive carcinomas; lane 9, high-grade invasive carcinoma metastatic to distant lymph nodes; M, 100-bp DNA ladder. *HGPRT* was used as an internal control. (**B**) Correlation between ΔNp63 mRNA/HGPRT mRNA ratio and p63 immunoreactivity in 35 urothelial tissue samples. ^*a*^Median and range. ^*b*^All samples with intense staining showed homogeneous (⩾50%) staining.

Next, we examined p63 mRNA expression profiles in normal and tumour tissues, using ΔNp63- and TAp63-specific primer sets by RT–PCR. Samples included three normal urothelial tissues, 31 primary tumours (10 LPN, four low-grade papillary T1, three high-grade papillary Ta, seven high-grade T1, and 7 muscle-invasive) and one metastatic lesion to the supraclavicular lymph nodes. Photographs of gels of the representative samples are shown in Figure 3A

.

In normal urothelial tissues, ΔNp63 mRNA was abundantly expressed while TAp63 mRNA was undetectable (Figure 3A). Tumour samples also expressed ΔNp63 mRNA at various levels whereas TAp63 mRNA was undetectable (Figure 3A). PCR amplification at 36 cycles for *TAp63* allowed detection of faint bands in approximately half of tumour samples, but the amounts of PCR products were not associated with pathologic phenotypes of the tumours (data not shown). Consistent with the results of immunostaining, eight of the 10 LPN tumours expressed ΔNp63 mRNA, while only two of eight muscle-invasive or metastatic carcinomas were positive for ΔNp63 mRNA. Median and range of the ΔNp63 mRNA/HGPRT mRNA ratio was 1.40 (1.37–1.55) for normal urothelium, 0.74 (0–1.65) for LPN tumour, 0.60 (0–1.67) for T1 tumour, and 0.00 (0–0.95) for muscle-invasive or metastatic carcinomas. In general, the ΔNp63 mRNA level was not increased in urothelial neoplasms compared with normal urothelium, indicating that ΔNp63 overexpression is not associated with urothelial tumorigenesis. The ΔNp63 mRNA/HGPRT mRNA ratio was significantly correlated with immunoreactivity (*r*_s_=0.56, *P*<0.01 by Spearman's rank correlation test; Figure 3B). Considering that the staining patterns with the Ab-1 antibody are virtually identical to those with the 4A4 antibody, ΔNp63 isoforms account for p63 protein expressed in normal and neoplastic urothelial tissues.

### Expression of p63 in cultured human normal and neoplastic urothelial cells

**Figure 4:**
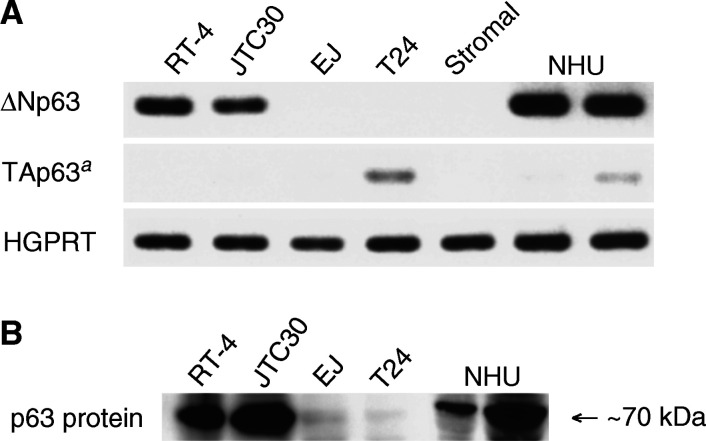
Expression of p63 in cultured cells. (**A**) Agarose gel electrophoresis of RT–PCR products using isoform-specific primer sets. ^*a*^As PCR products were undetectable at 30 cycles of PCR amplification, 40 cycles were performed for TAp63. (**B**) Immunoblotting for p63 protein using the 4A4 antibody.

We also examined p63 expression profiles in cultured normal and neoplastic urothelial cells. ΔNp63 mRNA was abundantly expressed in NHU cells but undetectable in stromal cells (Figure 4A

), confirming that p63 expression was confined to the urothelial compartment. For tumour cells, ΔNp63 mRNA was strongly expressed in low-grade cells (RT-4 and JTC30), whereas undetectable in high-grade aggressive cells (EJ and T24) (Figure 4A). Relative amounts of ΔNp63 mRNA to HGPRT mRNA for NHU, RT-4, and JTC30 cells were 1.29, 1.00, and 0.76, respectively. Expression of TAp63 mRNA was undetectable at 30 cycles of PCR amplification and barely detectable at 40 cycles (Figure 4A).

Immunoblot analysis of cell lysates also demonstrated major immunoreactive bands at approximately 70 kDa in NHU, RT-4, and JTC30 cells (Figure 4B), but not in stromal, EJ, and T24 cells. According to published data, candidates of p63 isoforms responsible for this specific band are TAp63*α*, TAp63*β*, and ΔNp63*α* (Yang *et al*, 1998; Hibi *et al*, 2000). Owing to the predominant mRNA expression of ΔN isoforms rather than TA isoforms in normal urothelial cells and low-grade urothelial tumour cells, our results suggest that ΔNp63*α* is the predominant isoform in these cell lines. These results are consistent with published data in human keratinocytes (Yang *et al*, 1998) and human prostate basal cells (Signoretti *et al*, 2000).

### Expression of p63 is not associated with p53 status

Since wild-type p53 protein mediates degradation of ΔNp63 *in vitro* (Ratovitski *et al*, 2001) and actually *p53* mutations strongly relate to overexpression of ΔNp63 in squamous cell carcinoma (Hibi *et al*, 2000), we investigated the possible association of p53 status with impaired ΔNp63 expression in urothelial carcinoma. The p53 status was assessed by immunohistochemistry using the PAb1801 antibody (Esrig *et al*, 1993; Koga *et al*, 2000). As shown in Table 2

**Table 2:** Association between p53 status and p63 expression in high-grade urothelial carcinomas

	**p63 immunoreactivity**		
**p53 immunoreactivity (*n*)**	**Intense**	**Weak**	**None**
Positive (14)	3	8	3
Negative (24)	5	15	4

*P*=0.93 by the *χ*^2^ test.

, we could not find any relation between p63 expression and p53 status in 38 high-grade urothelial carcinoma samples (six primary CIS, nine high-grade T1, and 23 muscle-invasive disease). This finding is consistent with another study on urothelial tumours (Park *et al*, 2000).

### Impaired p63 expression is associated with reduced *β*-catenin expression

A recent *in vitro* study has shown that ΔNp63 plays a role in regulating *β*-catenin levels (Patturajan *et al*, 2002). Therefore, we investigated the possible association of p63 expression status and *β*-catenin expression pattern. Of the 47 tumour samples examined, 29 (62%) showed homogeneously positive *β*-catenin staining at the cell membrane as observed in normal urothelium (Figure 1I). The remaining 18 (38%) showed reduced expression (Figure 1J), while positive nuclear staining was rare (4%, two out of 47). We could not find a statistically significant association between pathologic phenotypes and reduced *β*-catenin expression (4 out of 15 LPN tumours, two out of nine high-grade T1, and 12 out of 23 muscle-invasive carcinomas; *P*=0.15). Interestingly, impaired ΔNp63 expression is significantly associated with reduced *β*-catenin expression (*P*<0.01, Table 3

**Table 3:** Association between *β*-catenin expression and p63 expression in urothelial neoplasms

	**p63 immunoreactivity**		
***β*-Catenin expression (*n*)**	**Intense**	**Weak**	**None**
Normal (29)	16	12	11
Reduced (18)	2	11	5

*P*<0.01 by the *χ*^2^ test.

).

## DISCUSSION

This is the first study in which p63 expression has been systematically investigated in normal human urothelium and urothelial tumours of various pathologic phenotypes. Our results demonstrate distinctive p63 expression patterns according to phenotypic variants of urothelial neoplasms. First, a majority of LPN tumours expressed p63 in basal to middle cell layers as observed in normal urothelium, while CIS and high-grade/muscle-invasive carcinomas showed a frequent decrease or loss of p63 expression. Second, ΔNp63 isoforms account for p63 protein expressed in normal urothelium and urothelial neoplasms. Decreased expression of ΔNp63 is a common feature in high-grade invasive urothelial carcinomas, indicating that urothelial carcinogenesis is distinct from the squamous cell carcinoma transformation strongly associated with overexpression of ΔNp63 (Parsa *et al*, 1999; Hibi *et al*, 2000). Finally, we found a significant association of the impaired ΔNp63 expression with reduced *β*-catenin expression that relates to a worse prognosis of bladder cancer patients (Garcia del Muro *et al*, 2000; Shimazui *et al*, 1996).

Recently, experimental studies on transgenic mice have provided strong evidences that different genetic defects are responsible for the two distinctive pathways of urothelial tumorigenesis (Zhang *et al*, 1999,^2001^). Activation of Ha-*ras* alone, which plays a central role in mitogenic signal transduction, induces urothelial hyperplasia and papillary noninvasive tumours but not invasive carcinomas (Zhang *et al*, 2001), indicating that increased cell proliferation alone can lead to development of the papillary noninvasive urothelial tumour. On the other hand, inactivation of p53 and pRb by SV40 large T antigen induces CIS, invasive carcinoma, and metastatic disease (Zhang *et al*, 1999). These data imply that papillary noninvasive urothelial tumours have hyperplastic nature and are substantially different from aggressive carcinomas. The distinct patterns of p63 expression between LPN tumours and invasive carcinomas in the current study further strengthen the difference in the biological characters of these two phenotypes of urothelial neoplasms. Evaluation of p63 may be a useful tool for identifying tumours with highly malignant potential in their early phase.

In normal urothelia, p63 expression was strong in basal to intermediate cell layers but undetectable in umbrella cells, the terminally differentiated urothelial cells. This expression pattern closely resembles that observed in the epidermis; p63 expression is highest in keratinocyte stem cells (Pellegrini *et al*, 2001) and gradually decreases along the differentiation axis (Parsa *et al*, 1999; Bamberger *et al*, 2002). The *p63* knockout mice show severe skin defects (Mills *et al*, 1999; Yang *et al*, 1999), and remnant cells on the defected skin express terminal differentiation markers (Yang *et al*, 1999; Yang and McKeon, 2000), indicating physiological roles of p63 in sustaining the stem cell population and regulating differentiation of the epidermis. The urothelium also suffers from severe defects in the *p63*^−/−^ mice (Yang *et al*, 1999). Taken together, it appears likely that p63 plays critical roles in maintaining normal urothelial structure and regulating differentiation of the normal urothelium.

LPN tumours and normal urothelial cells of the basal cell compartment showed essentially the same p63 expression profiles. These data suggest that LPN tumours share common biological characters with normal urothelium. Considering the possible roles of p63 in the normal urothelium, LPN tumours might also require p63 expression to maintain their structure. Interestingly, the gradual diminution of p63 expression towards the superficial cell layers (Figure 1C) implies that the normal epithelial differentiation axis is still preserved in LPN tumours. Since LPN tumours actually remain confined to the epithelial layers and have little propensity for invasion and metastasis (Lee and Droller, 2000), it is controversial to classify this phenotypic variant as true carcinoma (Epstein *et al*, 1998). Our data may further support that LPN tumours are close to normal epithelium and have little potential of malignant progression.

High-grade invasive carcinomas, in clear contrast to LPN tumours, showed frequent decrease or loss of p63 expression. The mechanisms underlying impaired expression of p63 remain to be elucidated. Reportedly, mutations of the *p63* gene are rare in human primary tumours including urothelial tumours (Osada *et al*, 1998; Hagiwara *et al*, 1999; Sunahara *et al*, 1999; Park *et al*, 2000). In addition, we demonstrated that p53 status did not affect p63 expression in urothelial carcinomas, as previously reported (Park *et al*, 2000). Taken together, impaired ΔNp63 expression in urothelial carcinomas appears to be ascribed to epigenetic modification independent of the p53 pathway.

*β*-Catenin plays a dual role in cell–cell adherent junctions (AJs) and signal transduction that promotes cell proliferation (Conacci-Sorrell *et al*, 2002). Cancer invasion and metastasis could be promoted by not only disruption of AJs because of loss of E-cadherin or *α*-catenin or tyrosine phosphorylation of AJ components but also nuclear accumulation of *β*-catenin and consequent transactivation of its target genes (Conacci-Sorrell *et al*, 2002). Reduced expression of *β*-catenin that reflects defective AJs, on the other hand, has been demonstrated to associate with progression of urothelial carcinoma (Shimazui *et al*, 1996; Garcia del Muro *et al*, 2000). The latter process is rarely involved in urothelial neoplasms as demonstrated in the current study and by others (Stoehr *et al*, 2002; Zhu *et al*, 2000). Interestingly, diminished ΔNp63 was significantly associated with reduced *β*-catenin expression in the present study. Recently, Patturajan *et al*. have reported that downregulation of ΔNp63 decreases *β*-catenin levels *in vitro* in squamous cell carcinoma cells (Patturajan *et al*, 2002). Taken together, the impaired ΔNp63 expression might reduce *β*-catenin expression, which possibly associates with aggressive biological behaviour as well as malignant pathologic phenotypes of urothelial neoplasms. In fact, reduced *β*-catenin associates with a worse prognosis of bladder cancer patients (Shimazui *et al*, 1996; Garcia del Muro *et al*, 2000).

In conclusion, our data clearly demonstrate that the normal p63 expression pattern is highly preserved in LPN tumours, whereas frequently impaired in high-grade or invasive urothelial carcinomas. Moreover, impaired ΔNp63 expression associates with reduced *β*-catenin. These data suggest that the impaired ΔNp63 expression characterises biological aggressiveness of urothelial neoplasms.

## References

[bib1] Bamberger C, Pollet D, Schmale H (2002) Retinoic acid inhibits downregulation of delta Np63 alpha expression during terminal differentiation of human primary keratinocytes. J Invest Dermatol 118: 133–138 11851886 10.1046/j.0022-202x.2001.01649.x

[bib2] Brawn PN (1982) The origin of invasive carcinoma of the bladder. Cancer 50: 515–519 7093894 10.1002/1097-0142(19820801)50:3<515::aid-cncr2820500323>3.0.co;2-q

[bib3] Bubenik J, Baresova M, Viklicky V, Jakoubkova J, Sainerova H, Donner J (1973) Established cell line of urinary bladder carcinoma (T24) containing tumor-specific antigen. Int J Cancer 11: 765–773 4133950 10.1002/ijc.2910110327

[bib4] Cairns P, Proctor AJ, Knowles MA (1991) Loss of heterozygosity at the RB locus is frequent and correlates with muscle invasion in bladder carcinoma. Oncogene 6: 2305–2309 1766677

[bib5] Celli J, Duijf P, Hamel BC, Bamshad M, Kramer B, Smits AP, Newbury-Ecob R, Hennekam RC, Van Buggenhout G, van Haeringen A, Woods CG, van Essen AJ, de Waal R, Vriend G, Haber DA, Yang A, McKeon F, Brunner HG, van Bokhoven H (1999) Heterozygous germline mutations in the p53 homolog p63 are the cause of EEC syndrome. Cell 99: 143–153 10535733 10.1016/s0092-8674(00)81646-3

[bib6] Conacci-Sorrell M, Zhurinsky J, Ben-Ze'ev A (2002) The cadherin–catenin adhesion system in signaling and cancer. J Clin Invest 109: 987–991 11956233 10.1172/JCI15429 PMC150951

[bib7] Di Como CD, Urist MJ, Babayan I, Drobnjak M, Hedvat CV, Teruya-Feldstein J, Pohar K, Hoos A, Cordon-Cardo C (2002) p63 expression profiles in human normal and tumor tissues. Clin Cancer Res 8: 494–501 11839669

[bib8] Epstein JI, Amin MB, Reuter VR, Mostofi FK, Committee tBCC (1998) The World Health Organization/International Society of Urological Pathology Consensus classification of urothelial (transitional cell) neoplasms of the bladder. Am J Surg Pathol 22: 1435–1448 9850170 10.1097/00000478-199812000-00001

[bib9] Esrig D, Spruck CH, Nichols PW, Chaiwun B, Steven K, Groshen S, Chen S, Skinner DG, Jones PA, Cote RJ (1993) p53 nuclear protein accumulation correlates with mutations in the p53 gene, tumor grade, and stage in bladder cancer. Am J Pathol 143: 1389–1397 7901994 PMC1887166

[bib10] Evans DR, Irwin RJ, Harve PA, Bouchard JG, Kato T, Prout GRJ (1977) The activity of the pyrimidine biosynthetic pathway in MGH-U1 transitional cell carcinoma cells growth in tissue culture. J Urol 127: 712–719 10.1016/s0022-5347(17)58598-5 875145

[bib11] Garcia del Muro X, Torregrosa A, Munoz J, Castellsague X, Condom E, Vigues F, Arance A, Fabra A, Germa JR (2000) Prognostic value of the expression of E-cadherin and beta-catenin in bladder cancer. Eur J Cancer 36: 357–362 10708937 10.1016/s0959-8049(99)00262-2

[bib12] Grossman HB (1996) Superficial bladder cancer: decreasing the risk of recurrence. Oncology (Huntingt) 10: 1617–1624, discussion 1624, 1627–1628 8953583

[bib13] Hagiwara K, McMenamin MG, Miura K, Harris CC (1999) Mutational analysis of the p63/p73L/p51/p40/CUSP/KET gene in human cancer cell lines using intronic primers. Cancer Res 59: 4165–4169 10485447

[bib14] Hastings RJ, Franks LM (1983) Cellular heterogeneity in a tissue culture cell line derived from a human bladder carcinoma. Br J Cancer 47: 233–244 6572066 10.1038/bjc.1983.31 PMC2011288

[bib15] Hibi K, Trink B, Patturajan M, Westra WH, Caballero OL, Hill DE, Ratovitski EA, Jen J, Sidransky D (2000) AIS is an oncogene amplified in squamous cell carcinoma. Proc Natl Acad Sci USA 97: 5462–5467 10805802 10.1073/pnas.97.10.5462 PMC25851

[bib16] Hudson MA, Herr HW (1995) Carcinoma *in situ* of the bladder. J Urol 153: 564–572 7861485 10.1097/00005392-199503000-00002

[bib17] Kakuya T, Yamada T, Yokokawa M, Ueda T (1983) Establishment of cell strains from human urothelial carcinoma and their morphological characterization. In Vitro 19: 591–599 6684098 10.1007/BF02619572

[bib18] Kawakami S, Arai G, Hayashi T, Fujii Y, Xia G, Kageyama Y, Kihara K (2002) Peroxisome proliferator-activated receptor gamma ligands suppress proliferation of human urothelial basal cells *in vitro*. J Cell Physiol 191: 310–319 12012326 10.1002/jcp.10099

[bib19] Kawakami S, Fujii Y, Winters SJ (2001a) Follistatin production by skin fibroblasts and its regulation by dexamethasone. Mol Cell Endocrinol 172: 157–167 11165049 10.1016/s0303-7207(00)00371-3

[bib20] Kawakami S, Kageyama Y, Fujii Y, Kihara K, Oshima H (2001b) Inhibitory effect of *N*-acetylcysteine on invasion and MMP-9 production of T24 human bladder cancer cells. Anticancer Res 21: 213–219 11299737

[bib21] Koga F, Kitahara S, Arai K, Honda M, Sumi S, Yoshida K (2000) Negative p53/positive p21 immunostaining is a predictor of favorable response to chemotherapy in patients with locally advanced bladder cancer. Jpn J Cancer Res 91: 416–423 10804290 10.1111/j.1349-7006.2000.tb00961.x PMC5926463

[bib22] Lee R, Droller MJ (2000) The natural history of bladder cancer. Implications for therapy. Urol Clin North Am 27: 1–13, vii 10696240 10.1016/s0094-0143(05)70229-9

[bib23] Mills AA, Zheng B, Wang XJ, Vogel H, Roop DR, Bradley A (1999) p63 is a p53 homologue required for limb and epidermal morphogenesis. Nature 398: 708–713 10227293 10.1038/19531

[bib24] Mostofi FK (1973) Histological Typing of Urinary Bladder Tumours. Geneva: World Health Organization

[bib25] Orlow I, Lacombe L, Hannon GJ, Serrano M, Pellicer I, Dalbagni G, Reuter VE, Zhang ZF, Beach D, Cordon-Cardo C (1995) Deletion of the p16 and p15 genes in human bladder tumors. J Natl Cancer Inst 87: 1524–1529 7563186 10.1093/jnci/87.20.1524

[bib26] Osada M, Ohba M, Kawahara C, Ishioka C, Kanamaru R, Katoh I, Ikawa Y, Nimura Y, Nakagawara A, Obinata M, Ikawa S (1998) Cloning and functional analysis of human p51, which structurally and functionally resembles p53. Nat Med 4: 839–843 9662378 10.1038/nm0798-839

[bib27] Park BJ, Lee SJ, Kim JI, Lee CH, Chang SG, Park JH, Chi SG (2000) Frequent alteration of p63 expression in human primary bladder carcinomas. Cancer Res 60: 3370–3374 10910040

[bib28] Parsa R, Yang A, McKeon F, Green H (1999) Association of p63 with proliferative potential in normal and neoplastic human keratinocytes. J Invest Dermatol 113: 1099–1105 10594758 10.1046/j.1523-1747.1999.00780.x

[bib29] Patturajan M, Nomoto S, Sommer M, Fomenkov A, Hibi K, Zangen R, Poliak N, Califano J, Trink B, Ratovitski E, Sidransky D (2002) DeltaNp63 induces beta-catenin nuclear accumulation and signaling. Cancer Cell 1: 369–379 12086851 10.1016/s1535-6108(02)00057-0

[bib30] Pellegrini G, Dellambra E, Golisano O, Martinelli E, Fantozzi I, Bondanza S, Ponzin D, McKeon F, De Luca M (2001) p63 identifies keratinocyte stem cells. Proc Natl Acad Sci USA 98: 3156–3161 11248048 10.1073/pnas.061032098 PMC30623

[bib31] Ratovitski EA, Patturajan M, Hibi K, Trink B, Yamaguchi K, Sidransky D (2001) p53 associates with and targets Delta Np63 into a protein degradation pathway. Proc Natl Acad Sci USA 98: 1817–1822 11172034 10.1073/pnas.98.4.1817 PMC29340

[bib32] Rigby CC, Franks LM (1970) A human tissue culture cell line from a transitional cell tumor of a urinary bladder: growth, chromosome pattern and ultrastructure. Br J Cancer 24: 746–754 5503601 10.1038/bjc.1970.89 PMC2008730

[bib33] Rollnick BR, Hoo JJ (1988) Genitourinary anomalies are a component manifestation in the ectodermal dysplasia, ectrodactyly, cleft lip/palate (EEC) syndrome. Am J Med Genet 29: 131–136 3278611 10.1002/ajmg.1320290116

[bib34] Schalken JA, van Moorselaar RJ, Bringuier PP, Debruyne FM (1992) Critical review of the models to study the biologic progression of bladder cancer. Semin Surg Oncol 8: 274–278 1462097 10.1002/ssu.2980080505

[bib35] Schmale H, Bamberger C (1997) A novel protein with strong homology to the tumor suppressor p53. Oncogene 15: 1363–1367 9315105 10.1038/sj.onc.1201500

[bib36] Senoo M, Seki N, Ohira M, Sugano S, Watanabe M, Inuzuka S, Okamoto T, Tachibana M, Tanaka T, Shinkai Y, Kato H (1998) A second p53-related protein, p73L, with high homology to p73. Biochem Biophys Res Commun 248: 603–607 9703973 10.1006/bbrc.1998.9013

[bib37] Shimazui T, Schalken JA, Giroldi LA, Jansen CF, Akaza H, Koiso K, Debruyne FM, Bringuier PP (1996) Prognostic value of cadherin-associated molecules (alpha-, beta-, and gamma-catenins and p120cas) in bladder tumors. Cancer Res 56: 4154–4158 8797585

[bib38] Signoretti S, Waltregny D, Dilks J, Isaac B, Lin D, Garraway L, Yang A, Montironi R, McKeon F, Loda M (2000) p63 is a prostate basal cell marker and is required for prostate development. Am J Pathol 157: 1769–1775 11106548 10.1016/S0002-9440(10)64814-6 PMC1885786

[bib39] Sobin LH, Wittekind CH (1997) UICC-TNM Classification of Malignant Tumours. New York: Wiley-Liss

[bib40] Spruck III, CH, Ohneseit PF, Gonzalez-Zulueta M, Esrig D, Miyao N, Tsai YC, Lerner SP, Schmutte C, Yang AS, Cote R (1994) Two molecular pathways to transitional cell carcinoma of the bladder. Cancer Res 54: 784–788 8306342

[bib41] Steinberg GD, Trump DL, Cummings KB (1992) Metastatic bladder cancer. Natural history, clinical course, and consideration for treatment. Urol Clin North Am 19: 735–746 1279877

[bib42] Stoehr R, Krieg RC, Knuechel R, Hofstaedter F, Pilarsky C, Zaak D, Schmitt R, Hartmann A (2002) No evidence for involvement of beta-catenin and APC in urothelial carcinomas. Int J Oncol 20: 905–911 11956582

[bib43] Sunahara M, Shishikura T, Takahashi M, Todo S, Yamamoto N, Kimura H, Kato S, Ishioka C, Ikawa S, Ikawa Y, Nakagawara A (1999) Mutational analysis of p51A/TAp63gamma, a p53 homolog, in non-small cell lung cancer and breast cancer. Oncogene 18: 3761–3765 10391684 10.1038/sj.onc.1202972

[bib44] Trink B, Okami K, Wu L, Sriuranpong V, Jen J, Sidransky D (1998) A new human p53 homologue. Nat Med 4: 747–748 9662346 10.1038/nm0798-747

[bib45] Yang A, Kaghad M, Wang Y, Gillett E, Fleming MD, Dotsch V, Andrews NC, Caput D, McKeon F (1998) p63, a p53 homolog at 3q27–29, encodes multiple products with transactivating, death-inducing, and dominant-negative activities. Mol Cell 2: 305–316 9774969 10.1016/s1097-2765(00)80275-0

[bib46] Yang A, McKeon F (2000) P63 and P73: P53 mimics, menaces and more. Nat Rev Mol Cell Biol 1: 199–207 11252895 10.1038/35043127

[bib47] Yang A, Schweitzer R, Sun D, Kaghad M, Walker N, Bronson RT, Tabin C, Sharpe A, Caput D, Crum C, McKeon F (1999) p63 is essential for regenerative proliferation in limb, craniofacial and epithelial development. Nature 398: 714–718 10227294 10.1038/19539

[bib48] Zhang ZT, Pak J, Huang HY, Shapiro E, Sun TT, Pellicer A, Wu XR (2001) Role of Ha-*ras* activation in superficial papillary pathway of urothelial tumor formation. Oncogene 20: 1973–1980 11360181 10.1038/sj.onc.1204315

[bib49] Zhang ZT, Pak J, Shapiro E, Sun TT, Wu XR (1999) Urothelium-specific expression of an oncogene in transgenic mice induced the formation of carcinoma *in situ* and invasive transitional cell carcinoma. Cancer Res 59: 3512–3517 10416618

[bib50] Zhu X, Kanai Y, Saito A, Kondo Y, Hirohashi S (2000) Aberrant expression of beta-catenin and mutation of exon 3 of the beta-catenin gene in renal and urothelial carcinomas. Pathol Int 50: 945–952 11123760 10.1046/j.1440-1827.2000.01139.x

